# Parotid Mucoepidermoid Carcinoma in a Pediatric Patient: A Case Report

**DOI:** 10.7759/cureus.54609

**Published:** 2024-02-21

**Authors:** Claudia C Gonzalez, Lisamelia Espaillat, David Rosario, Massiel P Gonzalez

**Affiliations:** 1 Medicine, Pontificia Universidad Catolica Madre y Maestra, Santiago, DOM; 2 Otolaryngology - Head and Neck Surgery, Hospital Metropolitano de Santiago, Santiago, DOM; 3 Radiology, Diagnostica Social, La Romana, DOM

**Keywords:** salivary gland neoplasm, case report, female pediatric patient, parotid gland, mucoepidermoid carcinoma

## Abstract

Mucoepidermoid carcinomas (MECas) are malignant epithelial salivary gland neoplasms composed of a variable mixture of epidermoid and mucus-secreting cells arising from the ductal epithelium. Of all salivary gland tumors, MECas are the most common malignant lesions of the parotid gland. This case report aims to present a 14-year-old female patient with a history of progressive enlargement of a 3 cm in diameter, painless, mobile mass located at the parotid gland without facial nerve dysfunction. The lesion was exhaustively studied preoperatively, and studies were carried out. Contrast-enhanced computerized tomography (CECT) showed an increase in nodule numbers and size at the level of both the jugular and posterior cervical chains. In contrast, the gland's fine needle aspiration (FNA) showed a mucinous background. The histologic depiction established that the tumor was MECa of the parotid gland. The literature reviews on MECa encompass discussions about its prevalence, etiology, histological findings, and treatment.

## Introduction

Mucoepidermoid carcinomas (MECas) are malignant epithelial salivary gland neoplasms that are composed of a variable mixture of both epidermoid and mucus-secreting cells that arise from the ductal epithelium [1]. Of all salivary gland tumors, MECas encompass nearly 30% of all salivary gland malignancies and are the most common malignant lesions of the parotid gland, with 60% occurring there [1-3]. These are the most commonly occurring pediatric salivary gland malignancies, with the literature stating that 35% of salivary gland neoplasms in the pediatric population are malignant, of which 60% are MECas [1, 2]. The most associated signs that MECas of the parotid gland exhibit include a palpable parotid mass that tends to be rock hard, facial pain, otalgia, and facial nerve paralysis [1]. 

Furthermore, MECas can be graded from low to high, and the prognosis will vary depending on the grade. Low-grade MECas have a 6% local recurrence rate and usually a 90% 10-year survival rate. On the other hand, high-grade MECas have a 78% local recurrence and only a 27% 10-year survival rate [1]. The occurrence of MECas affects women more frequently than men, with a ratio of 3:2, and the mean age onset is usually in the fifth decade of life [4]. A possible etiology for MECas appears to be radiation exposure [5, 6]. Differential diagnoses for MECas of the parotid gland include parotid benign mixed tumors, Warthin tumors, parotid adenoid cystic carcinomas, parotid non-Hodgkin lymphomas, parotid metastatic nodes, and parotid ductal carcinomas [1]. Other differential diagnoses, not exclusive to the parotid gland, would include necrotizing sialometaplasia (palate), mucocele, inverted papilloma or cystadenoma, cystadenocarcinoma, primary or metastatic squamous cell carcinoma, and low-grade polymorphous adenocarcinoma [7, 8].

## Case presentation

We present a 14-year-old, relatively healthy female patient without a significant medical family history who arrived at the otorhinolaryngology consult with a history of painless swelling in the right infra-auricular region of the parotid gland and progressive growth with one year of evolution. Upon physical examination, there was a nodular lesion in the right parotid region of approximately 3 cm in diameter, painless, mobile, and with intact facial nerve function. 

**Figure 1:**
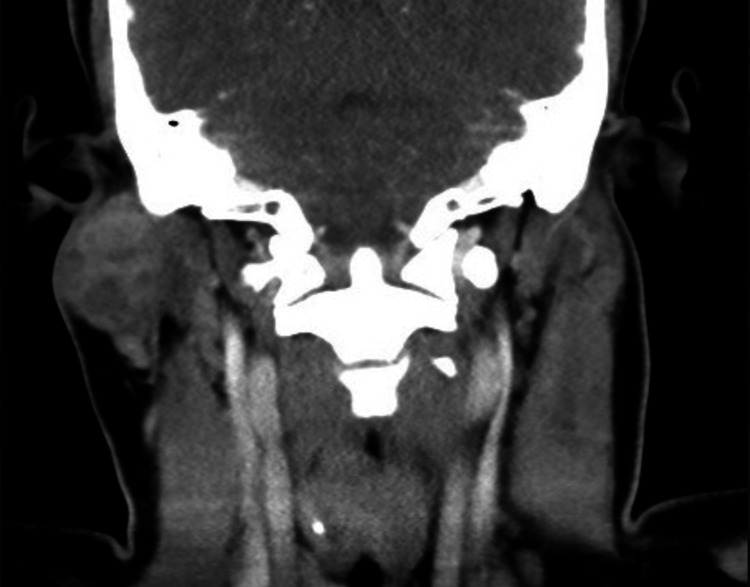
Contrast-enhanced computed tomography (axial image) After administering the contrast medium, a slight enhancement of the lesion was identified, better delimiting the areas with cystic degeneration.

**Figure 2:**
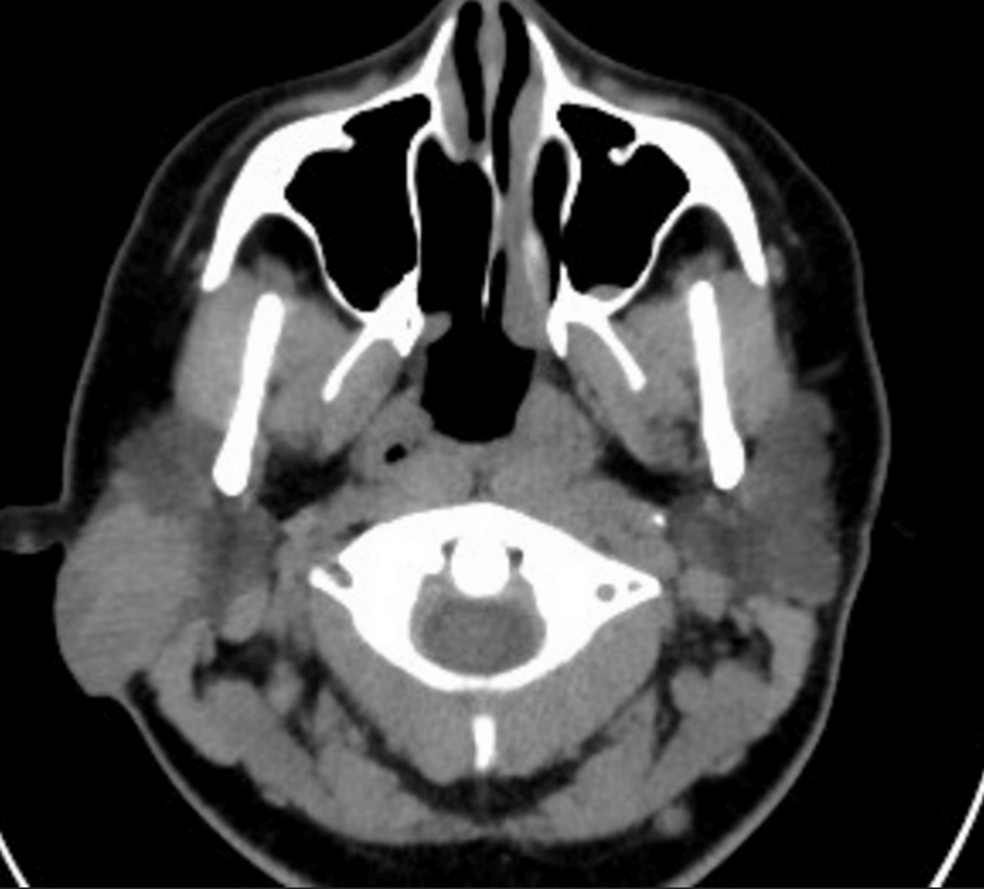
Contrast-enhanced computed tomography (axial image) An expansive lesion was identified located in the parotid gland in the superficial lobe, which was isodense to the muscle, had hypodense areas and smooth edges, and presented an extension to the subcutaneous cellular tissue.

A high-dose contrast-enhanced computed tomography (CECT) (Figure 1) showed multiple lymph nodes increased in number and size at the level of both jugular chains and posterior cervical, the largest ones measuring approximately 1.6 x 1.2 cm in right zone III of the neck. A retention cyst found in the left maxillary sinus measured approximately 2.2 cm. Additionally, an expansive lesion in the superficial lobe of the right parotid gland, which measured approximately 3.4 x 2.8 x 2.7 cm, presented with multiple nodules and rounded cystic images inside (Figure 2). After the administration of the contrast medium, it presented a slight heterogeneous enhancement. Moreover, the fine needle aspiration (FNA) of the parotid gland biopsy revealed a mucinous background. An epithelial proliferation made up of mucus-secreting cells without atypia, rounded nuclei without frank atypia, and ample cytoplasm with mucoid content was observed, which was suggestive of a low-grade mucoepidermoid carcinoma. 

**Figure 3:**
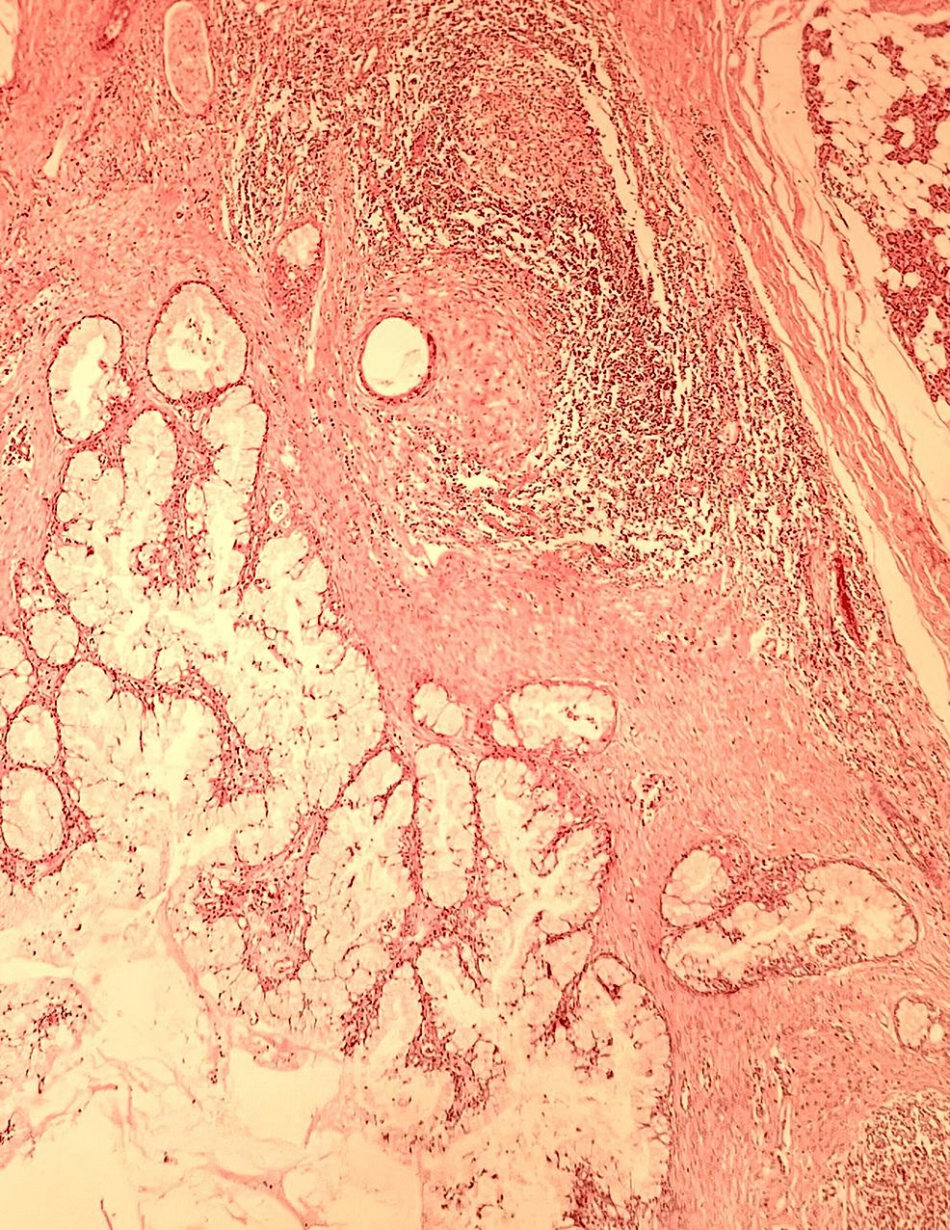
Hematoxylin and eosin (H&E) stain The salivary gland was observed with areas of mucinous and focal squamoid differentiation.

**Figure 4:**
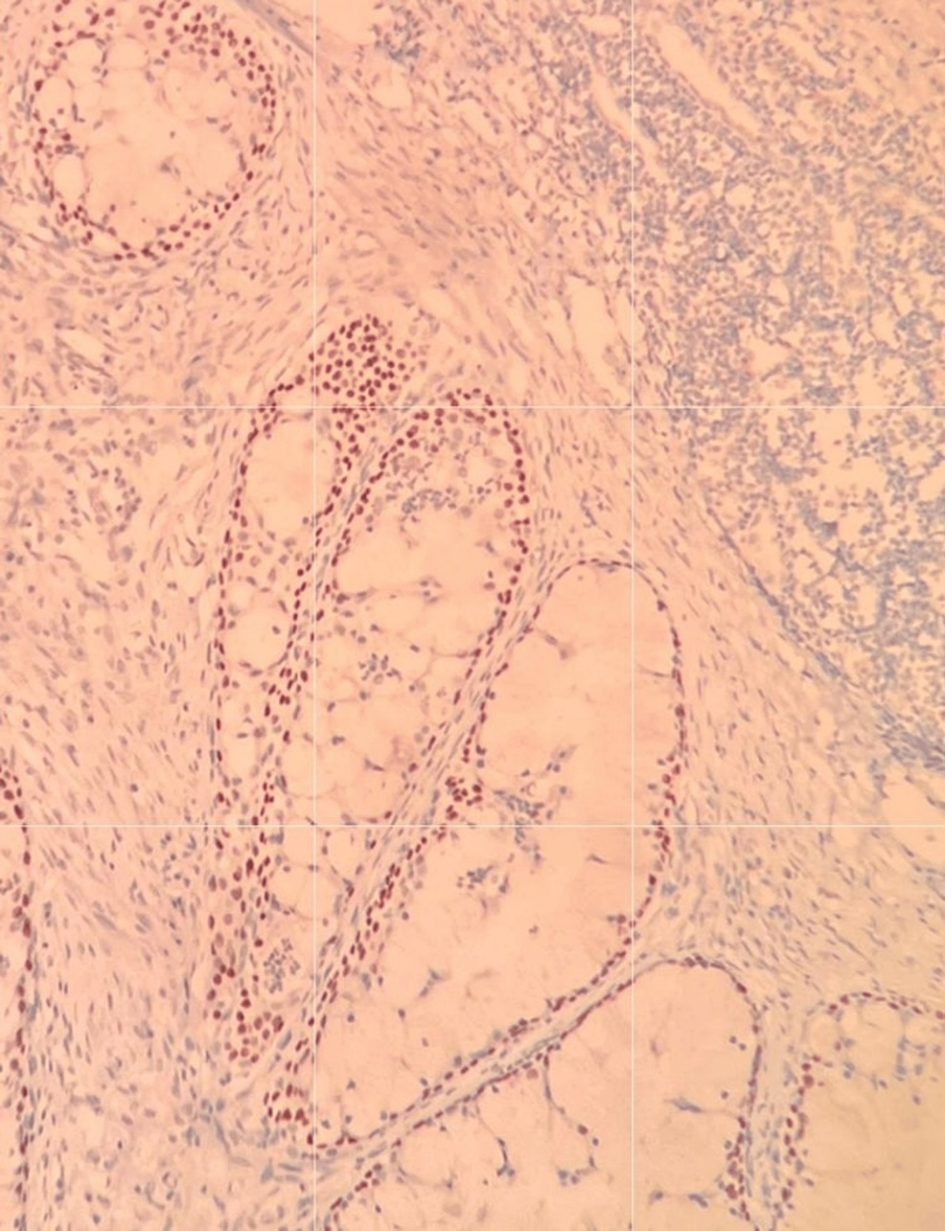
Positive nuclear immunostaining for p63 in mucoepidermoid carcinoma of the salivary gland

**Figure 5:**
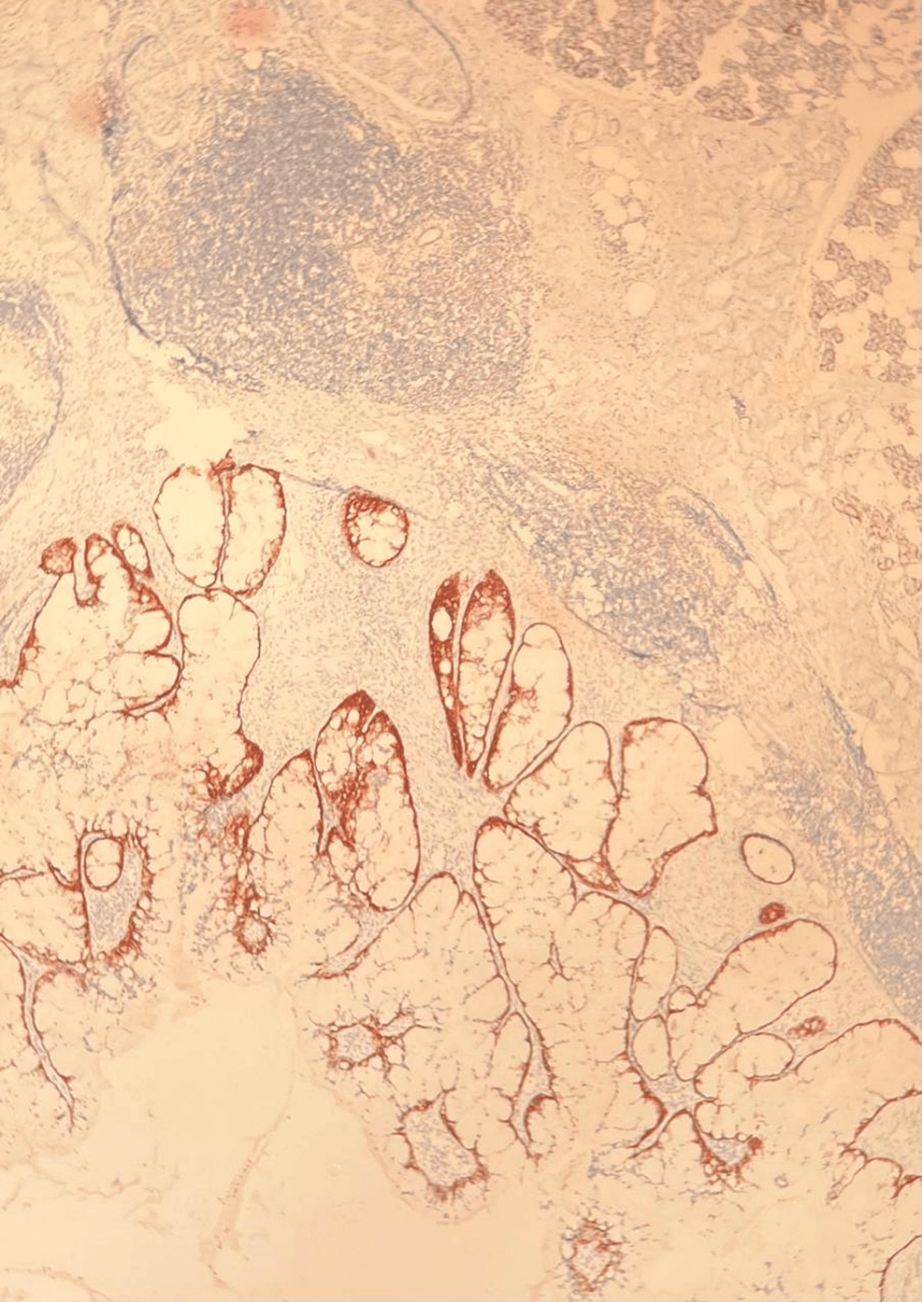
Positive cytoplasmic immunostaining for EMA in mucoepidermoid carcinoma of the salivary gland EMA: epithelial membrane antigen

The product of resection of the right parotid gland was confirmed to be a low-grade mucoepidermoid carcinoma measuring 3 x 2.5 x 2.2 cm, which had no evidence of extraparenchymal extension without necrosis (Figures 3-5). It also had a moderate inflammatory reaction and did not have lymphatic or perineural vascular invasion. The salivary gland was preserved, and the skin was not affected. Surgical margins were negative for malignancy, whereas the neck area biopsy revealed reactive chronic lymphadenitis. The tumor, node, metastasis (TNM) staging of the lesion was T2N0M0.

**Figure 6:**
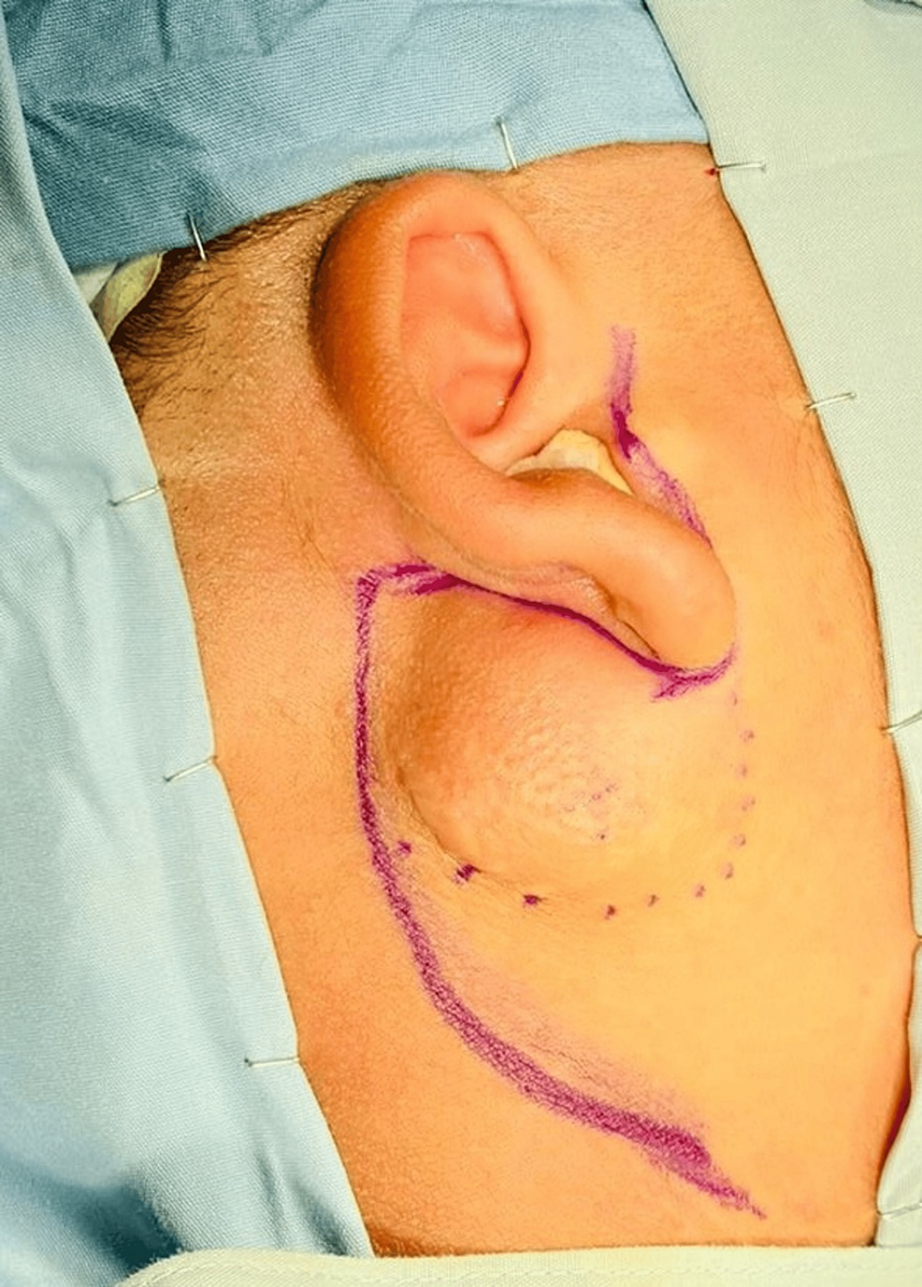
Pre-surgical markings before superficial parotidectomy

**Figure 7:**
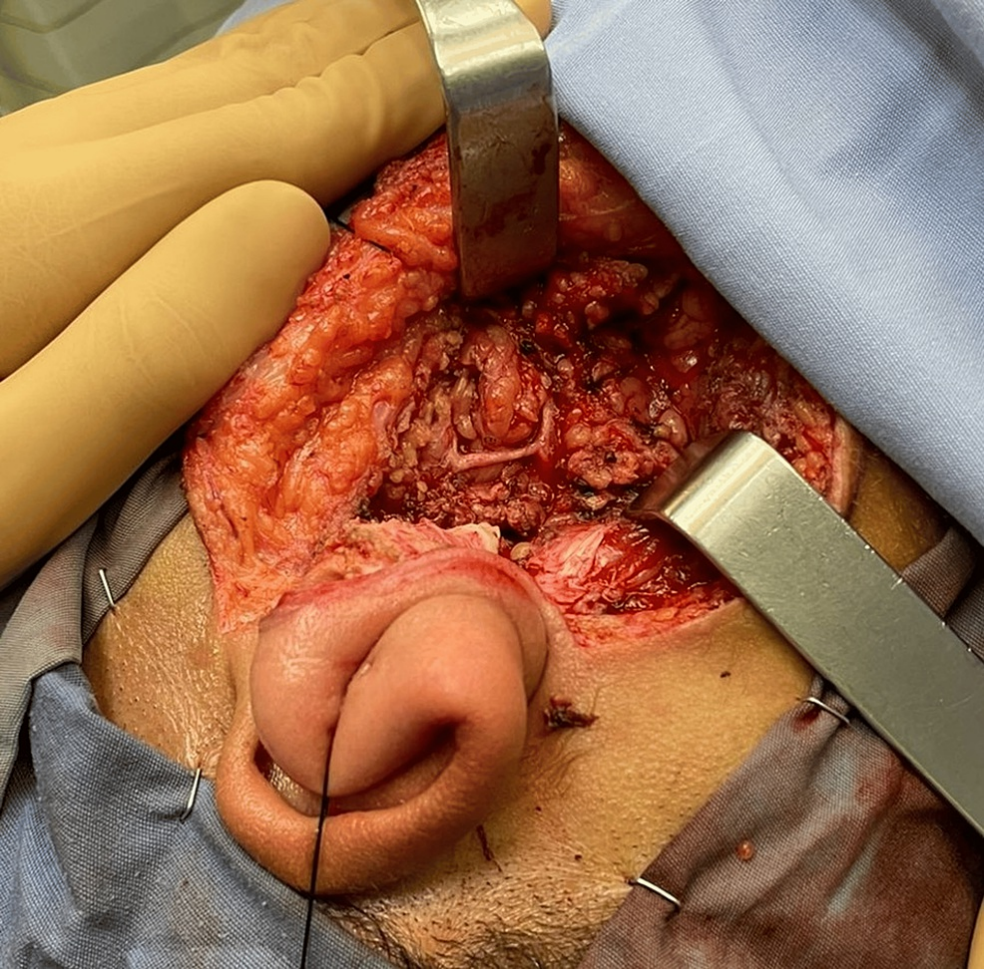
Intraoperative photograph demonstrating excision of the tumor and preservation of the facial nerve.

After a pre-surgical evaluation, the patient was scheduled for surgery. Since it was already confirmed by the CECT and biopsy that it was a low-grade mucoepidermoid carcinoma, a superficial parotidectomy was performed with preservation of the facial nerve (Figures 6-7). The patient, consequently, was referred to clinical oncology, where a PET scan was prescribed to evaluate the requirement for postoperative radiotherapy. 

Follow-up and outcomes

**Figure 8:**
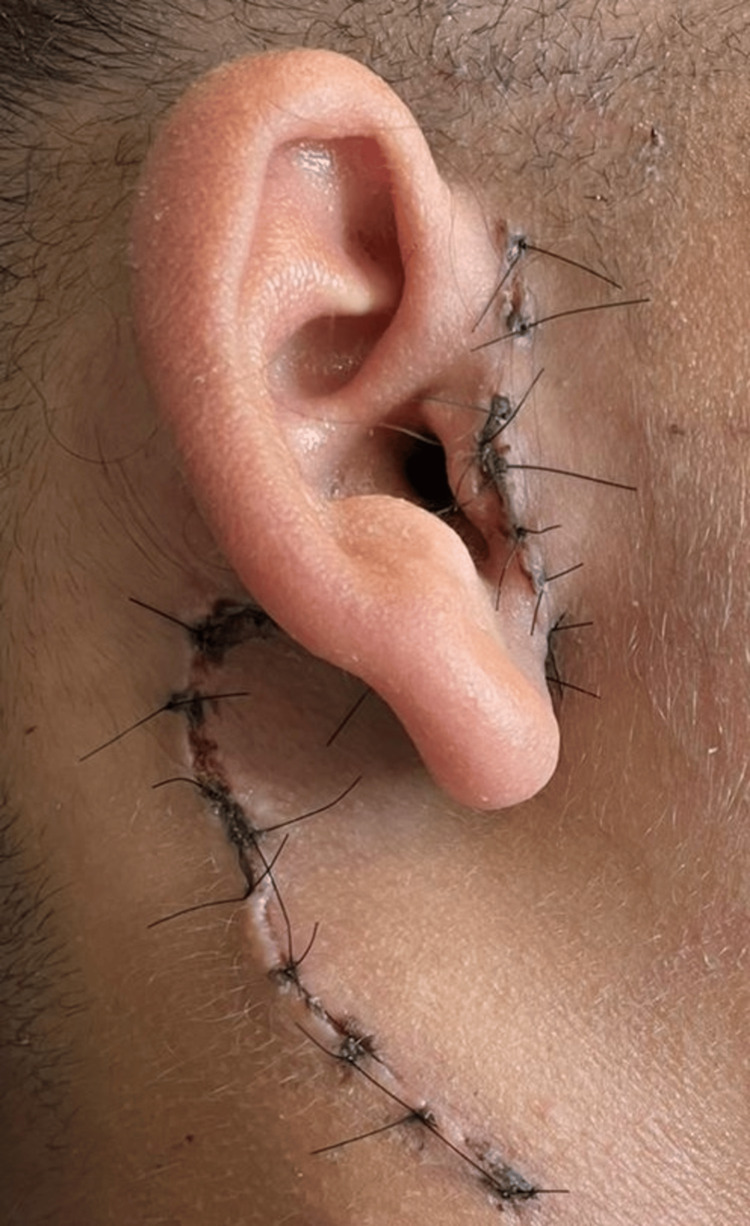
Postoperative image at the one-week follow-up

**Figure 9:**
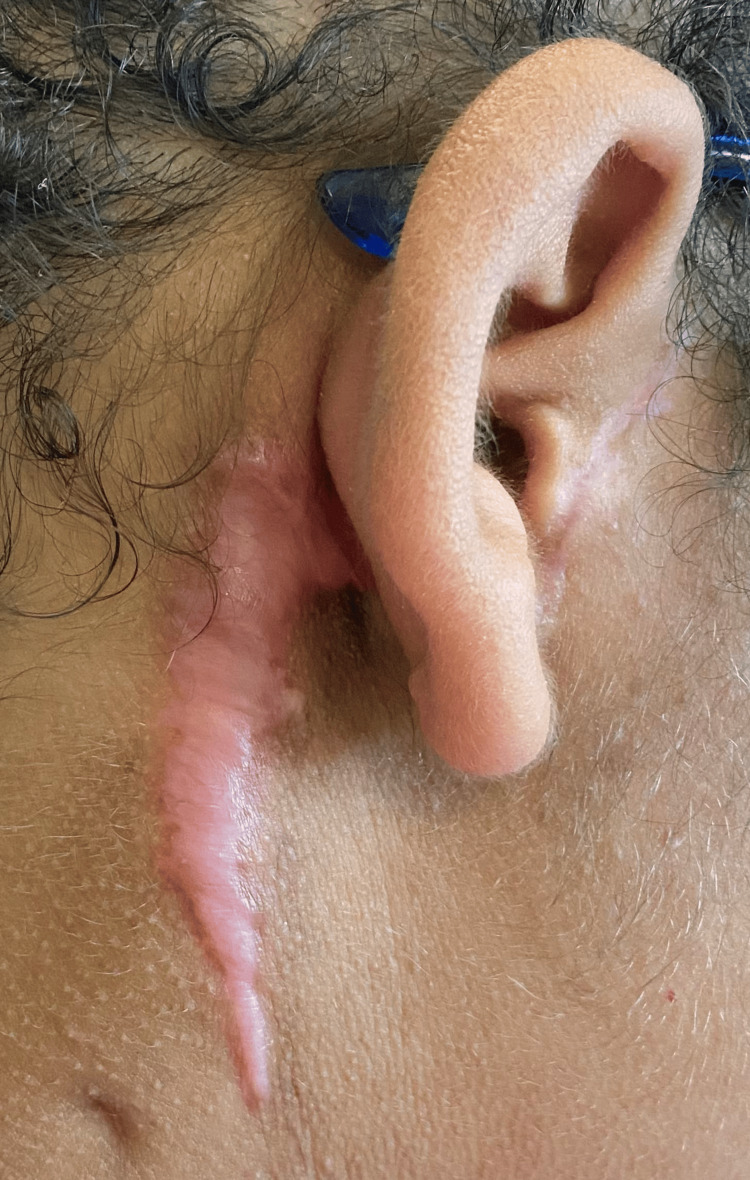
At the two-month postoperative follow-up, a scar with keloid formation is seen.

Follow-up was done one week after the surgery (Figure 8), and then again in one month. The patient was able to recover well. After two months of follow-up, it was observed that there was keloid formation in the scar (Figure 9). A corticosteroid solution was applied twice in 20 days directly into the scar to prevent further formation of keloids.

## Discussion

Mucoepidermoid carcinoma is the most common type of malignant salivary gland tumor; approximately 35% of two to three individuals per 100,000 individuals each year get diagnosed, which demonstrates its rare occurrence [2,5,9]. These types of salivary gland neoplasms tend to be reported more often in persons belonging to the middle-aged group; however, it is the most frequent malignant salivary gland tumor in the pediatric population, in addition to being moderately more common in female patients [1,5,10]. At the genetic level, approximately 80% of MECas seem to have a distinct ​​t(11;19)(q21;p13) translocation with a CRTC1(MECT1)-MAML2 fusion [11, 12]; however, in the case of our patient, genetic evaluations were not performed as they are not routinely done. Some authors believe, however, that the MAML2 gene arrangement should not be used as a prognostic marker for MECas [12]. Common signs associated with MECas of the parotid gland consist of a palpable parotid mass that tends to be rock hard, facial pain, otalgia, and facial nerve paralysis [1].

At the histopathological level, the classification of MECas involves three grades: low, intermediate, and high. Low-grade tumors, constituting 48% of cases, are more prevalent than high-grade tumors (38.7%), with intermediate-grade tumors being the least common (13.3%) [13]. These grades are determined based on factors such as the degree of cytological atypia, the extent of cyst formation, and the relative abundance of mucous, epidermoid, and intermediate cells [14]. Tumors with a low grade exhibit a higher proportion of mucous cells and are considered less aggressive, while high-grade tumors, characterized by a lower mucous cell ratio, are deemed more malignant and associated with a poorer prognosis [13]. Furthermore, low-grade MECas are well-circumscribed with a varied parotid space mass, while high-grade MECas will be invasive with a vague parotid space mass association with cancerous nodes. Clinical presentation will also depend on the MECa grade, where low-grade tumors will present with a painless and mobile but slowly growing mass; high-grade MECas, however, will present with a tender, immobile, and rapidly growing mass. Treatment for low-grade MECa tumors includes wide local excision with preservation of the facial nerve, superficial parotidectomy if possible, and postoperative radiotherapy. However, total parotidectomy may be only necessary if the tumor involves the deep lobe of the parotid gland [1].

## Conclusions

Mucoepidermoid carcinomas are recognized as the most common malignant growths affecting the parotid gland in the pediatric population. Moreover, key associated indicators of parotid gland MECs include the presence of a firm and immovable mass in the parotid region, along with symptoms such as facial pain, earache, and facial nerve paralysis. The recommended treatment for MECs involves surgical removal, and the prognosis hinges on factors such as clinical stage, location, grade, and the effectiveness of the surgical procedure. Consequently, these tumors should be considered in the differential diagnosis of individuals presenting with parotid gland inflammation to ensure timely and accurate identification.
